# Emerging infectious diseases: a CDC perspective.

**DOI:** 10.3201/eid0707.017702

**Published:** 2001

**Authors:** J M Hughes

**Affiliations:** Centers for Disease Control and Prevention, Atlanta, Georgia 30333, USA. jmh2@cdc.gov


### Emerging Infectious Diseases: A CDC Perspective

**James M. Hughes**
Centers for Disease Control and Prevention, Atlanta, Georgia, USA

Dr. Jeffrey Koplan, Director of the Centers for Disease Control and Prevention (CDC), has set four goals for the agency to accomplish. Each is directly related to issues being discussed at the 2000 ICEID conference. The first goal is to strengthen the science base for public health action. The second goal is to collaborate with healthcare partners for disease prevention; it is essential that individuals in clinical and academic medicine work closely with colleagues in public health to address these issues. The third goal is to promote healthy living for people at every stage of life. Finally, and very importantly to participants in this conference, the fourth goal for the agency is to work with partners to improve global health.

An article in Morbidity and Mortality Weekly Report in 1999 contains a summary of progress made in infectious disease control in the United States during the 20th century when the number of deaths resulting from infectious diseases decreased dramatically (1). However, the dramatic spike in the number of deaths from 1918 to 1919 resulting from the first of three influenza pandemics, is clearly evident (Figure 1). In addition, the number of deaths caused by infectious diseases increased between 1980 and 1995. Because of the excellent progress made against infectious diseases during much of the 20th century, many people felt that the problem of infectious diseases had been sufficiently addressed. Nearly 40 years ago, Sir MacFarlane Burnett wrote, "One can think of the middle of the twentieth century as the end of one of the most important social revolutions in history, the virtual elimination of the infectious disease as a significant factor in social life" (2). This quotation reveals the complacency that has existed since and goes a long way toward explaining why we have gotten behind both nationally and globally in terms of capacity required to deal with the problems of infectious diseases.

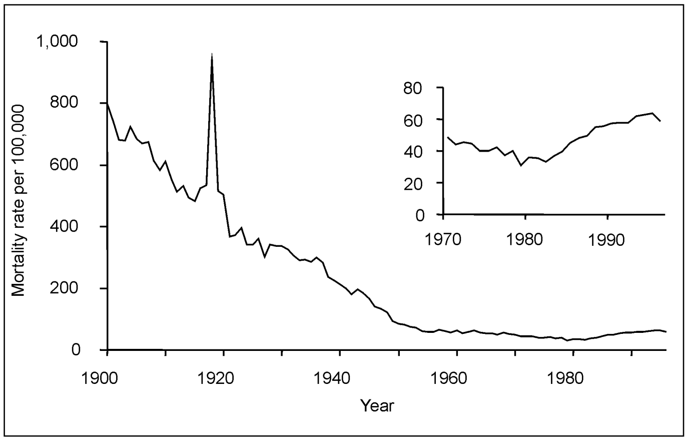

Figure 1. Trends in Infectious Diseases Mortality, 1900-1996. Deaths resulting from infectious diseases decreased markedly in the United States during most of the 20th century. However, between 1980 and 1992, the death rate from infectious diseases increased 58%. The sharp increase in infectious disease deaths in 1918 and 1919 was caused by an influenza pandemic, which killed more than 20 million people.

The current problems we face as a result were highlighted in a very important 1992 Institute of Medicine (IOM) report, Emerging Infections: Microbial Threats to Health in the United States (3). This seminal work represents the effort of an expert committee cochaired by Dr. Joshua Lederberg and Dr. Robert Shope. This committee defined emerging infections as "new, reemerging, or drug-resistant infections whose incidence in humans has increased within the past two decades or whose incidence threatens to increase in the near future."

The committee also identified six major factors that contribute to disease emergence and reemergence: 1) changes in human demographics and behavior, 2) advances in technology and changes in industry practices, 3) economic development and changes in land-use patterns, 4) dramatic increases in volume and speed of international travel and commerce—movement not only of people but of animals, foodstuffs, and other commodities, 5) microbial adaptation and change (a factor that makes infectious diseases unique and particularly challenging), and 6) breakdown of public health capacity required for infectious diseases at the local, state, national, and global levels. In most cases, more than one of these factors are applicable to the emergence or reemergence of an individual disease or syndrome.

The IOM report contains 15 recommendations, many of which we felt were directed specifically to CDC. We responded to that report by developing a CDC Emerging Infections Plan, issued in 1994 (4), and an updated version, published in 1998 (5), that outlines a strategy for CDC to work with many partners throughout the country and around the world to address these issues. The plan contains four goals. The first emphasizes the need to strengthen infectious disease surveillance and response; this approach is necessary to ensure timely detection and control of diseases and their agents. Second, many research issues raised by these challenges need to be addressed. Third, the public health system is in urgent need of repair so that it can deal with these issues; the CDC strategy emphasizes the training needs associated with human resource development, an important goal of this conference. The final, ultimate goal stresses the need to strengthen prevention and control programs locally, nationally, and globally.

This conference has several dominant themes. The first is antimicrobial resistance. The IOM has maintained a strong

Address for correspondence: James Hughes, Centers for Disease Control and Prevention, 1600 Clifton Road, NE, Mailstop C12, Atlanta, GA 30333, USA; fax: 404-639-3039; e-mail: jmh2@cdc.gov interest in emerging infectious disease control by recently issuing a report by an ongoing forum on antimicrobial resistance (6). Foodborne disease and food safety is another prominent theme. A number of presentations include data from a national surveillance network, the National Molecular Subtyping Network for Foodborne Disease Surveillance (PulseNet, Figure 2), which represents the vision for modern infectious disease surveillance (7).

Electronic linkages of individuals at local, state, and national levels who are utilizing modern molecular epidemiologic techniques in public health laboratories are absolutely essential to ensure the rapid identification of emerging foodborne diseases. This approach needs to be expanded beyond foodborne disease and linked with healthcare facilities and clinical laboratories to integrate our infectious disease surveillance systems. PulseNet represents a partnership between CDC, the Food and Drug Administration, the U.S. Department of Agriculture, the Association of Public Health Laboratories, and many individual state public health laboratories throughout the country.

This conference also emphasizes the global nature and scope of infectious diseases. Another recent IOM report acknowledges this point and concludes, “Distinctions between domestic and international health problems are losing their usefulness and often are misleading” (8). Before 1999, West Nile virus had never been found in the Western Hemisphere, though it was a well-recognized cause of disease in Africa, Europe, and the Middle East. Recent experience reinforces the need to address not only surveillance of and capacity to respond to vectorborne diseases, but also the importance of research on infectious diseases that exist in other parts of the world.

In the 8 years since the IOM Emerging Infections report was published, CDC has collaborated with Dr. David Heymann and his colleagues at the World Health Organization (WHO), along with many other individuals in many countries around the world, to deal with a number of infectious disease outbreaks. Lessons from this experience consistently emphasize the importance of infectious disease surveillance, the ability to rapidly conduct an epidemiologic investigation, and the need for trained staff and modern laboratory facilities to diagnose these diseases accurately and rapidly. Many outbreaks have reminded us of the disruption of travel and commerce that can occur when local outbreaks have global implications.

The West Nile encephalitis outbreak reinforces these lessons: that all of us need to keep an open mind about possible causes of a particular infectious disease outbreak; that clinicians and public health workers need to collaborate closely; and that people involved in human medicine and human public health need to interact on a more regular basis with colleagues in veterinary medicine and veterinary public health. State public health veterinarians have an important role in this regard. The experience with West Nile encephalitis also highlights the necessity of developing public

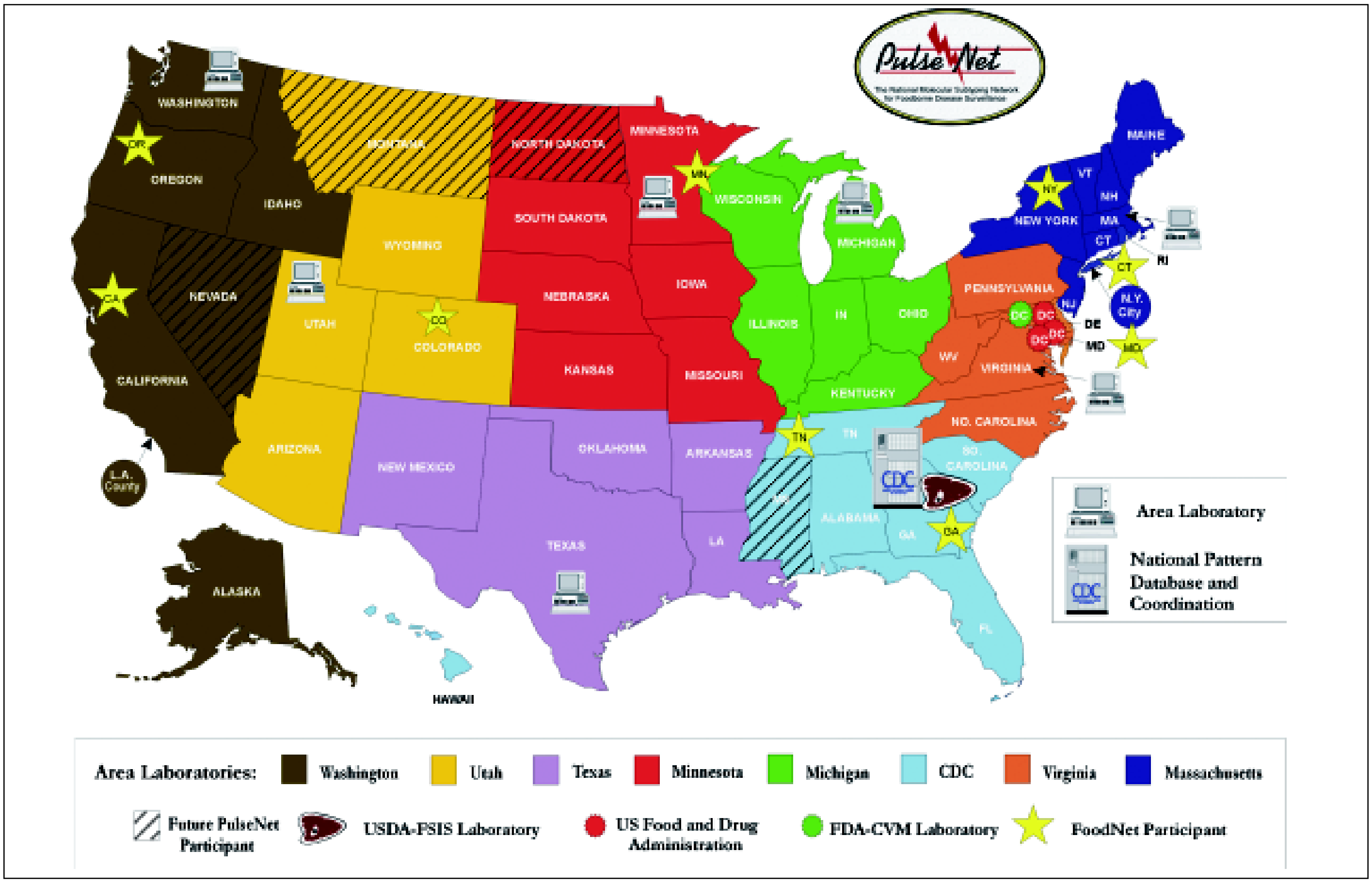

Figure 2. The National Molecular Subtyping Network for Foodborne Disease Surveillance. Area Lab Service and Support Zones.

health laboratory capacity and of continuing to invest in training of young people in disciplines such as entomology and wildlife biology, and it also reveals a number of critical communication issues that such outbreaks raise.

We have been very pleased during the past few years to work with the Association of Public Health Laboratories to increase CDC's role in training public health laboratory scientists. We have done this, in part, through an Emerging Infectious Diseases Laboratory Fellowship Program that initially had a domestic focus, but, with the support of Eli Lilly and Company and the CDC Foundation, now includes an international track to bring scientists from other countries to work with us at CDC or with colleagues in state public health department laboratories to acquire critical public health laboratory skills.

The West Nile virus outbreak also provides a vivid reminder that we need to consider the possibility that a complex infectious disease outbreak may result from bioterrorism. Preparing for this possibility will strengthen the national and global ability to address emerging and reemerging infections. In an address at the National Academy of Sciences in 1999, President Clinton said, "These cutting edge efforts (focused on bioterrorism preparedness) will address not only the threat of weapons of mass destruction but also the equally serious danger of emerging infectious diseases" (9). The future is hard to predict, but we can be pretty certain that we are going to continue to be challenged by the problem of antimicrobial resistance. We will eventually experience another influenza pandemic, and urban yellow fever threatens to reemerge in Latin America. Recent experience suggests that we will continue to need to deal with regional, national, and global outbreaks of foodborne disease. We are going to continue to be surprised by the range of chronic diseases that have infectious causes. Finally, we know that we are going to have to be prepared to confront the unexpected.

The intelligence community has acknowledged that infectious diseases represent a threat to national security (10). Leaders of the Group of Eight Industrialized Nations have made a commitment to substantially reduce the global burden of HIV infection, tuberculosis, and malaria by 2010 (11). This conference provides a timely opportunity for CDC and its many partners to examine lessons learned and review our commitment to rebuild national and global public health systems in order to address these three diseases as well as the numerous challenges posed by other emerging infectious diseases.

### References

1. Centers for Disease Control and Prevention. Control of infectious diseases. Morb Mortal Wkly Rep 1999;48:621-8.
2. Burnet M. Natural history of infectious disease. Cambridge: Cambridge University Press; 1962.
3. Institute of Medicine. Emerging infections: microbial threats to health in the United States. Washington: National Academy Press; 1992.
4. Centers for Disease Control and Prevention. Addressing emerging infectious disease threats: a prevention strategy for the United States. Atlanta: U.S. Department of Health and Human Services, Public Health Service; 1994.
5. Centers for Disease Control and Prevention. Preventing emerging infectious diseases: a strategy for the 21st century. Atlanta: U.S. Department of Health and Human Services, 1998.
6. IOM (Institute of Medicine). Antimicrobial resistance: issues and options. Workshop report: forum on emerging infections. Washington: National Academy Press; 1998.
7. Stephenson J. New approaches for detecting and curtailing foodborne microbial infections. JAMA 1997;277:1337-40.
8. IOM. America's vital interest in global health. Washington: National Academy Press; 1997.
9. Clinton WJ. Remarks by the President on keeping America secure for the 21st century. Washington DC: National Academy of Sciences; January 22, 1999.
10. National Intelligence Council. The global infectious disease threat and its implications for the United States. Washington: National Intelligence Estimate 99-17D; January 2000.
11. Watts J. Targets now set by G8 countries to reduce "diseases of poverty." Lancet 2000;356:408.
